# Fission-independent compartmentalization of mitochondria during budding yeast cell division

**DOI:** 10.1083/jcb.202211048

**Published:** 2024-01-05

**Authors:** Saori R. Yoshii, Yves Barral

**Affiliations:** 1 Department of Biology, Institute of Biochemistry, ETH Zürich, Zürich, Switzerland; 2 Department of Biochemistry and Molecular Biology, Graduate School and Faculty of Medicine, The University of Tokyo, Tokyo, Japan

## Abstract

Lateral diffusion barriers compartmentalize membranes to generate polarity or asymmetrically partition membrane-associated macromolecules. Budding yeasts assemble such barriers in the endoplasmic reticulum (ER) and the outer nuclear envelope at the bud neck to retain aging factors in the mother cell and generate naïve and rejuvenated daughter cells. However, little is known about whether other organelles are similarly compartmentalized. Here, we show that the membranes of mitochondria are laterally compartmentalized at the bud neck and near the cell poles. The barriers in the inner mitochondrial membrane are constitutive, whereas those in the outer membrane form in response to stresses. The strength of mitochondrial diffusion barriers is regulated positively by spatial cues from the septin axis and negatively by retrograde (RTG) signaling. These data indicate that mitochondria are compartmentalized in a fission-independent manner. We propose that these diffusion barriers promote mitochondrial polarity and contribute to mitochondrial quality control.

## Teaser

Yoshii and Barral discover that lateral diffusion barriers compartmentalize the membranes of yeast mitochondria into mother, bud, and tethered domains in a regulated manner, independent of the fission machinery.

## Introduction

In contrast to chromosomes, which segregate symmetrically, asymmetrically dividing cells partition cellular material such as organelles, extrachromosomal DNA, specific messenger RNA, and proteins unequally between daughter cells, endowing these with distinct identities. The budding yeast *Saccharomyces cerevisiae* undergoes asymmetric cell division where mother cells produce a limited number of daughter cells and eventually die, whereas their daughter cells are born rejuvenated, i.e., with a restored replicative lifespan (Denoth Lippuner et al., 2014) and naïve state (Caudron and Barral, 2013; Lau et al., 2022). This rejuvenation is achieved through the establishment of diffusion barriers at the bud neck in the endoplasmic reticulum (ER) and outer nuclear envelope membranes (Barral et al., 2000; Takizawa et al., 2000; Dobbelaere and Barral, 2004; Shcheprova et al., 2008; Luedeke et al., 2005; Clay et al., 2014). These barriers retain aging factors such as extrachromosomal ribosomal DNA (rDNA) circles, misfolded proteins, and memory traces in the mother (Shcheprova et al., 2008; Clay et al., 2014; Saarikangas et al., 2017; Baldi et al., 2017; Lau et al., 2022). While compartmentalization of the ER membrane is observed across many cell types from yeast to mammalian cells (Luedeke et al., 2005; Shcheprova et al., 2008; Lee et al., 2016; Moore et al., 2015; bin Imtiaz et al., 2022), whether diffusion barriers exist in other membranes has remained elusive.

Mitochondria form a dynamic network of tubules that undergo frequent fission and fusion events, resulting in the isolation or mixing of mitochondria of different qualities (Youle and van der Bliek, 2012). At any given time during the division cycle, the mitochondria of *S. cerevisiae* can be discontinuous (Higuchi-Sanabria et al., 2016; McFaline-Figueroa et al., 2011) or continuous (Jakobs et al., 2003) between the mother and bud. Accordingly, mitochondria frequently undergo fission and fusion throughout budding (Altmann et al., 2008; Jakobs et al., 2003). Although it has been known that material diffuses and is shared throughout continuous mitochondria (Youle and van der Bliek, 2012), whether lateral diffusion barriers constrain the dynamics of these exchanges has not been investigated. Here, we addressed this issue and characterized how mitochondrial proteins diffuse and exchange within the continuous mitochondria of budding yeast cells.

## Results

### The diffusion of the membrane proteins Tom20 and Atp1 but not that of soluble matrix proteins is restricted across continuous mitochondria

**Figure 1.**
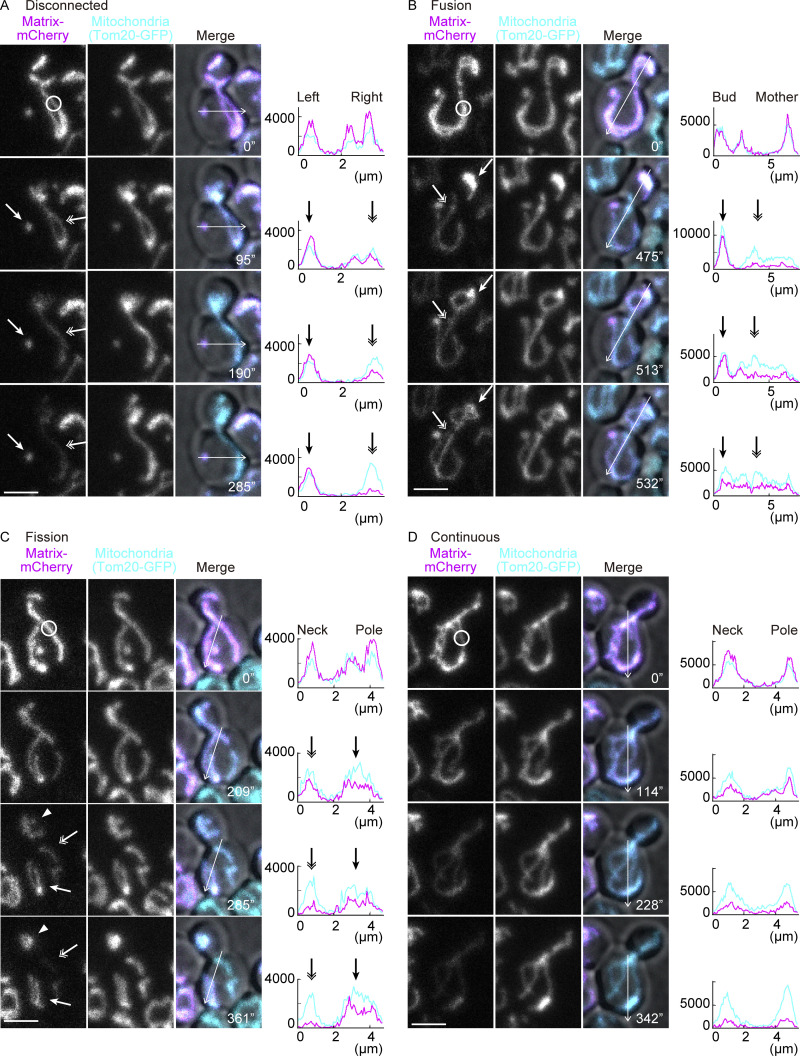
**Monitoring of mitochondrial continuity. (A–D)** mCherry FLIP in wild-type cells expressing Tom20-GFP (mitochondrial marker; without photobleach) and matrix-mCherry (photobleach target). The line graphs are the intensity profiles along the lines in the respective images. **(A)** Loss of matrix-mCherry serves as a continuity marker; a physically separate mitochondrion, indicated by an arrow, retained mCherry signals while a photobleached mitochondrion, indicated by a double arrow, lost the fluorescence. **(B)** An example of a fusion event. The photobleached mitochondrion in the mother (indicated by a double arrow) lost mCherry fluorescence while a physically separate mitochondrion in the bud (indicated by an arrow) retained the fluorescence, until these two mitochondria fused with each other, leading to equilibration of the mCherry fluorescence between the structures. The fusion event took place between 475 and 513 s. **(C)** An example of a fission event. A continuous mitochondrion was photobleached and lost mCherry fluorescence from the entire structure until it underwent fission forming three separate mitochondria. One mitochondrion at the bleaching area (indicated by a double arrow) further lost mCherry fluorescence whereas two separated mitochondria (indicated by an arrow and arrowhead) retained the fluorescence after fission. The fission event took place between 209 and 285 s. **(D)** An example of a continuous mitochondrion between the mother and bud throughout the imaging period. The mCherry fluorescence was lost from the entire structure. Photobleach was applied in the mCherry channel as indicated by white circles. Images are a sum projection of five z-stacks taken at 0.5-μm intervals. Scale bar: 3 µm.

**Figure 2.**
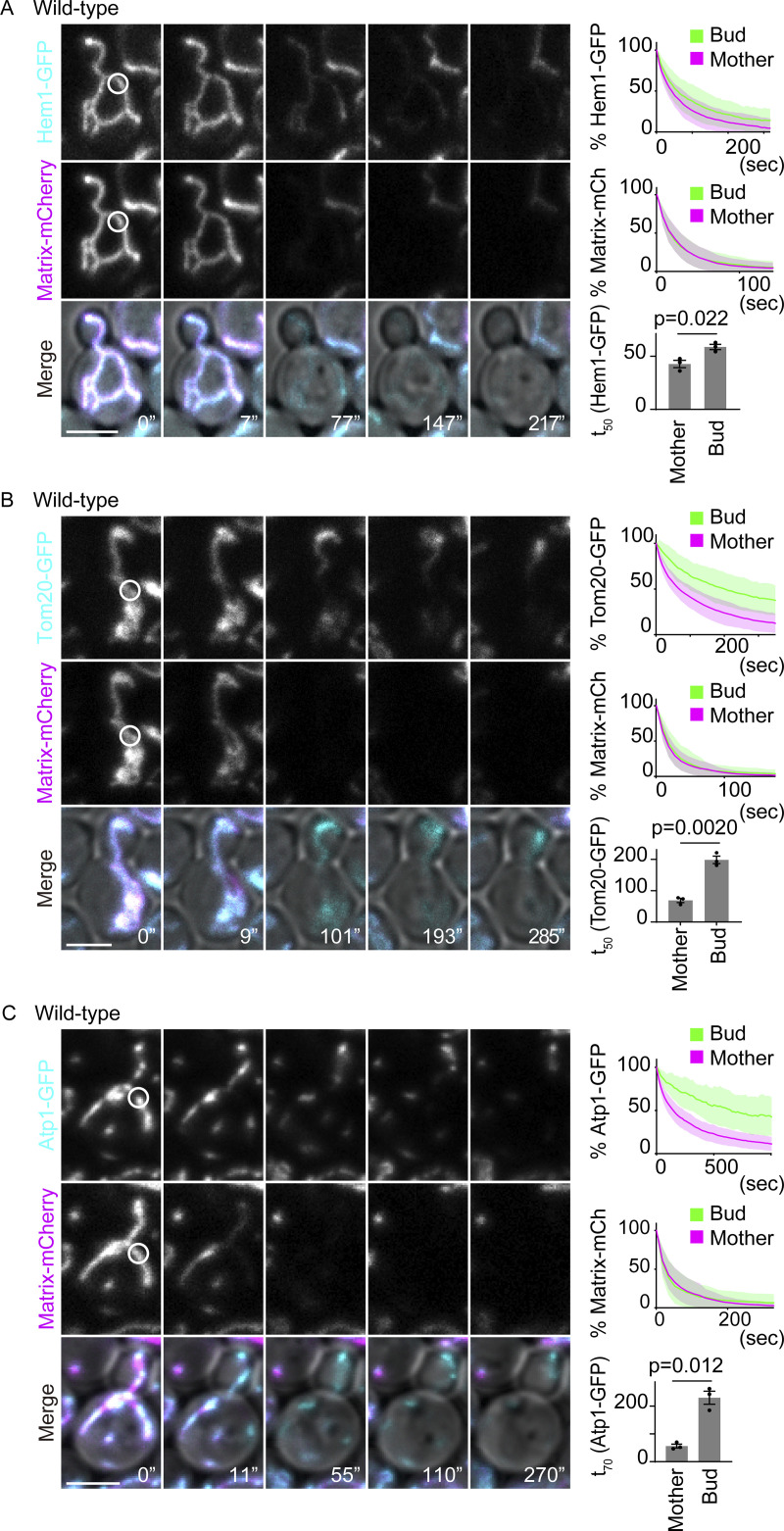
**Lateral compartmentalization of continuous mitochondria. (A–C)** Dual-color FLIP in wild-type cells expressing GFP-tagged mitochondrial proteins and matrix-mCherry. Representative images, pooled quantification data of GFP and mCherry FLIP, and t_X_ (time to reduce to X% of the total fluorescence) of GFP FLIP from three independent experiments are shown. **(A)** Wild-type cells expressing Hem1-GFP and matrix-mCherry (*n* = 30 cells, *n* = 10 cells for each experiment). **(B)** Wild-type cells expressing Tom20-GFP and matrix-mCherry (*n* = 32 cells, *n* ≥ 10 cells for each experiment). **(C)** Wild-type cells expressing Atp1-GFP and matrix-mCherry (*n* = 25 cells, *n* ≥ 8 cells for each experiment). Photobleach was applied in the GFP and mCherry channels as indicated with white circles. Images are a sum projection of five z-stacks taken at 0.5-μm intervals. Scale bar: 3 μm. Data from three independent clones were pooled to obtain the bleaching curves. Shadows represent mean ± SD. Error bar: mean ± SE. Welch’s two-tailed *t* test was applied to compare the t_X_ in the mother and bud.

**Figure S1.**
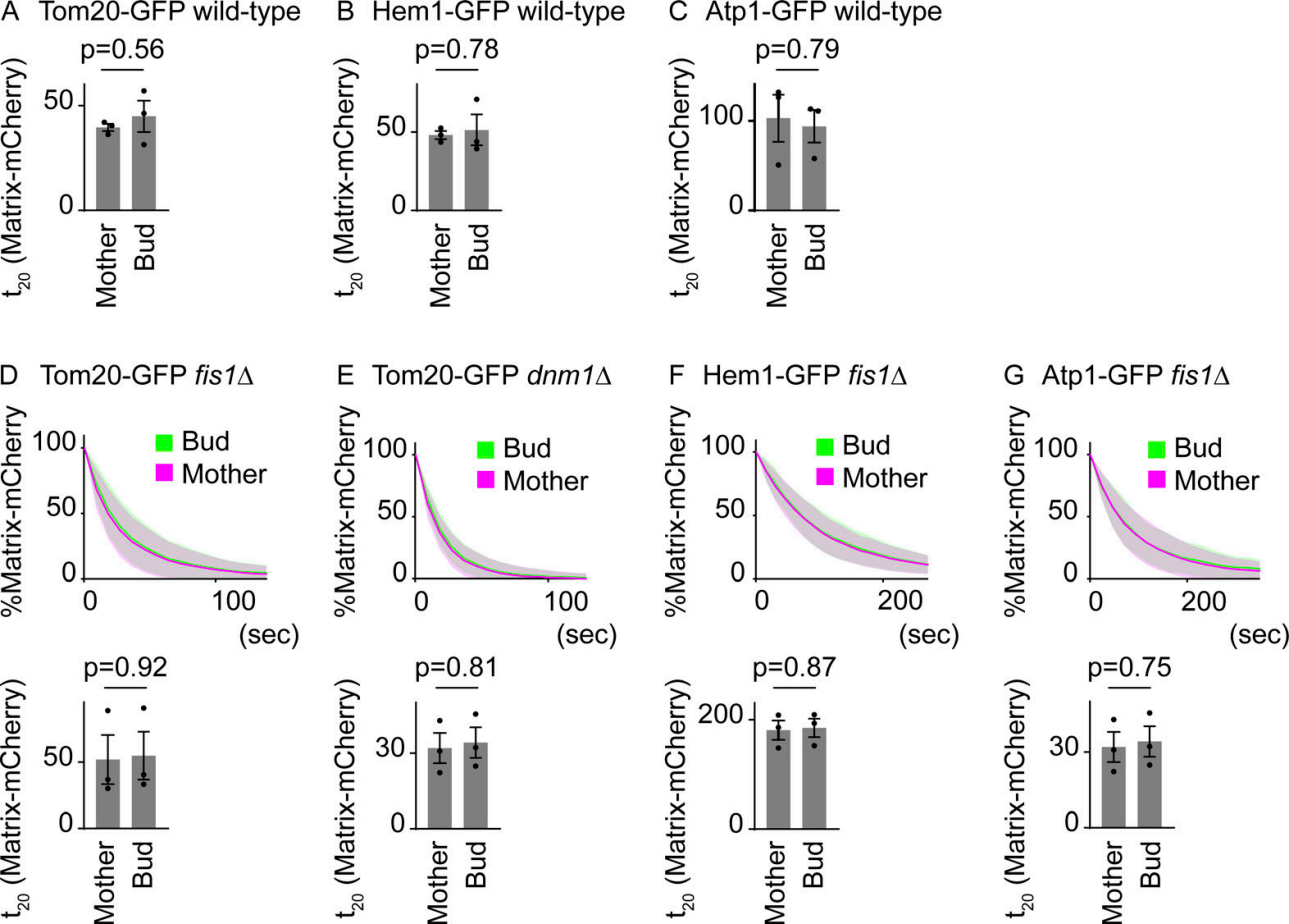
**Matrix-mCherry FLIP. (A–C)** t_20_ (time to reduce to 20% of the total fluorescence) of mCherry FLIP from three independent experiments are shown (mCherry data from Fig. 2). Note that t_X_ for mCherry cannot be directly compared with t_X_ for GFP due to the different bleaching conditions and different susceptibility to photobleaching. **(A)** Wild-type cells expressing Hem1-GFP and matrix-mCherry (*n* = 30 cells, *n* = 10 cells for each experiment). **(B)** Wild-type cells expressing Tom20-GFP and matrix-mCherry (*n* = 32 cells, *n* ≥ 10 cells for each experiment). **(C)** Wild-type cells expressing Atp1-GFP and matrix-mCherry (*n* = 25 cells, *n* ≥ 8 cells for each experiment). **(D–G)** Pooled quantification data of mCherry FLIP and t_20_ from three independent experiments are shown (mCherry data from Fig. 3, A–D). **(D)** *fis1*∆ cells expressing Tom20-GFP and matrix-mCherry (*n* = 32 cells, *n* ≥ 10 cells for each experiment). **(E)** *dnm1*∆ cells expressing Tom20-GFP and matrix-mCherry (*n* = 28 cells, *n* ≥ 7 cells for each experiment). **(F)** *fis1*∆ cells expressing Hem1-GFP and matrix-mCherry (*n* = 21 cells, *n* = 7 cells for each experiment). **(G)** *fis1*∆ cells expressing Atp1-GFP and matrix-mCherry (*n* = 25 cells, *n* ≥ 8 cells for each experiment). Data from three independent clones were pooled to obtain the bleaching curves. Shadows represent mean ± SD. Error bar: mean ± SE. Welch’s two-tailed *t* test was applied to compare the t_20_ in the mother and bud.

To determine whether continuous mitochondria constrain the flux of materials within themselves by being somehow sub-compartmentalized, we characterized the diffusion of mitochondrial proteins using dual-color fluorescence loss in photobleaching (FLIP) experiments. The green fluorescent protein (GFP) was fused to a mitochondrial protein of interest and the red fluorescent protein mCherry was expressed in the matrix (Westermann and Neupert, 2000). When photobleaching was applied in a small area of a mitochondrion, mCherry fluorescence was rapidly lost throughout the organelle. Concomitantly, this assay established, as expected, that separate mitochondria within the same cell did not exchange materials since mCherry fluorescence barely decayed in mitochondria disconnected from the bleached one (Fig. 1 A). As reported previously (Altmann et al., 2008; Jakobs et al., 2003), frequent fission and fusion events were observed. The fusion of two mitochondria resulted in rapid equilibration of matrix-mCherry signals (Fig. 1 B). In reverse, when mitochondria underwent fission, the residual signal stopped being bleached in the separated mitochondria (Fig. 1 C). Importantly, in mitochondria that spread out through the mother and bud, photobleaching anywhere in them caused the nearly simultaneous loss of mCherry fluorescence throughout them (Fig. 1 D). A similar pattern was observed for the soluble matrix protein Hem1 (Fig. 2 A and Fig. S1 A). These data indicate that soluble matrix proteins diffuse freely across and only across continuous mitochondria and established matrix-mCherry as a reliable indicator to assay mitochondrial continuity.

In contrast to matrix-mCherry, a GFP-tagged outer mitochondrial membrane (OMM) protein, Tom20, showed distinct fluorescence decay patterns in mitochondria that are continuous between the mother and bud. When photobleached locally in the mother, Tom20-GFP fluorescence decayed quickly throughout the mother part of the continuous mitochondrion but only slowly in the bud, suggesting that the exchange of Tom20 was restricted somewhere between the photobleached area and the bud part of the mitochondrion (Fig. 2 B and Fig. S1 B). The corresponding diffusion patterns were observed for the inner mitochondrial membrane (IMM) protein Atp1; fluorescence loss of Atp1-GFP was delayed in the bud upon photobleaching in the mother, indicating that its diffusion in the IMM is restricted (Fig. 2 C and Fig. S1 C). These data suggested that while soluble matrix proteins diffused freely within continuous mitochondria, the diffusion of mitochondrial membrane proteins was confined, indicating that the mitochondrial membranes might be laterally compartmentalized.

### Delayed diffusion of membrane proteins is independent of mitochondrial fission

**Figure 3.**
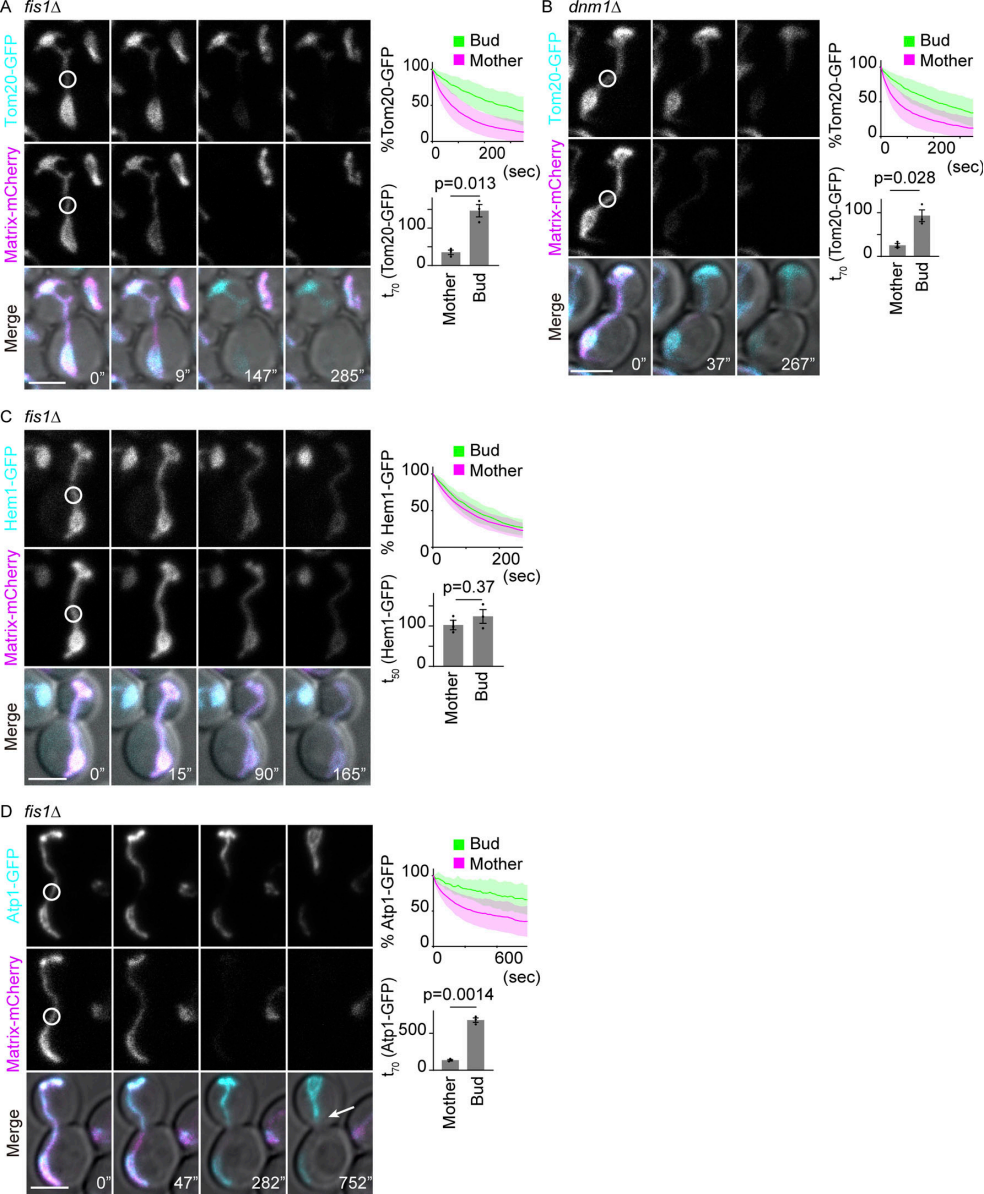
**Compartmentalization of mitochondria is independent of the fission machinery. (A–D)** Dual-color FLIP in *fis1*∆ cells expressing GFP-tagged mitochondrial proteins and matrix-mCherry. Representative images, pooled quantification data of GFP FLIP, and t_X_ of GFP FLIP from three independent experiments are shown. Photobleach was applied in the GFP and mCherry channels as indicated with white circles. (Note here that the bleaching and imaging conditions were changed and the graphs cannot be directly compared with Fig. 2). **(A)** *fis1*∆ cells expressing Tom20-GFP and matrix-mCherry (*n* = 32 cells, *n* ≥ 10 cells for each experiment). **(B)** *dnm1*∆ cells expressing Tom20-GFP and matrix-mCherry (*n* = 28 cells, *n* ≥ 7 cells for each experiment). **(C)** *fis1*∆ cells expressing Hem1-GFP and matrix-mCherry (*n* = 21 cells, *n* = 7 cells for each experiment). **(D)** *fis1*∆ cells expressing Atp1-GFP and matrix-mCherry (*n* = 25 cells, *n* ≥ 8 cells for each experiment). Images are a sum projection of five z-stacks taken at 0.5 μm intervals. Scale bar: 3 μm. Data from three independent clones were pooled to obtain the bleaching curves. Shadows represent mean ± SD. Error bar: mean ± SE. Welch’s two-tailed *t* test was applied to compare the t_X_ in the mother and bud.

**Figure 4.**
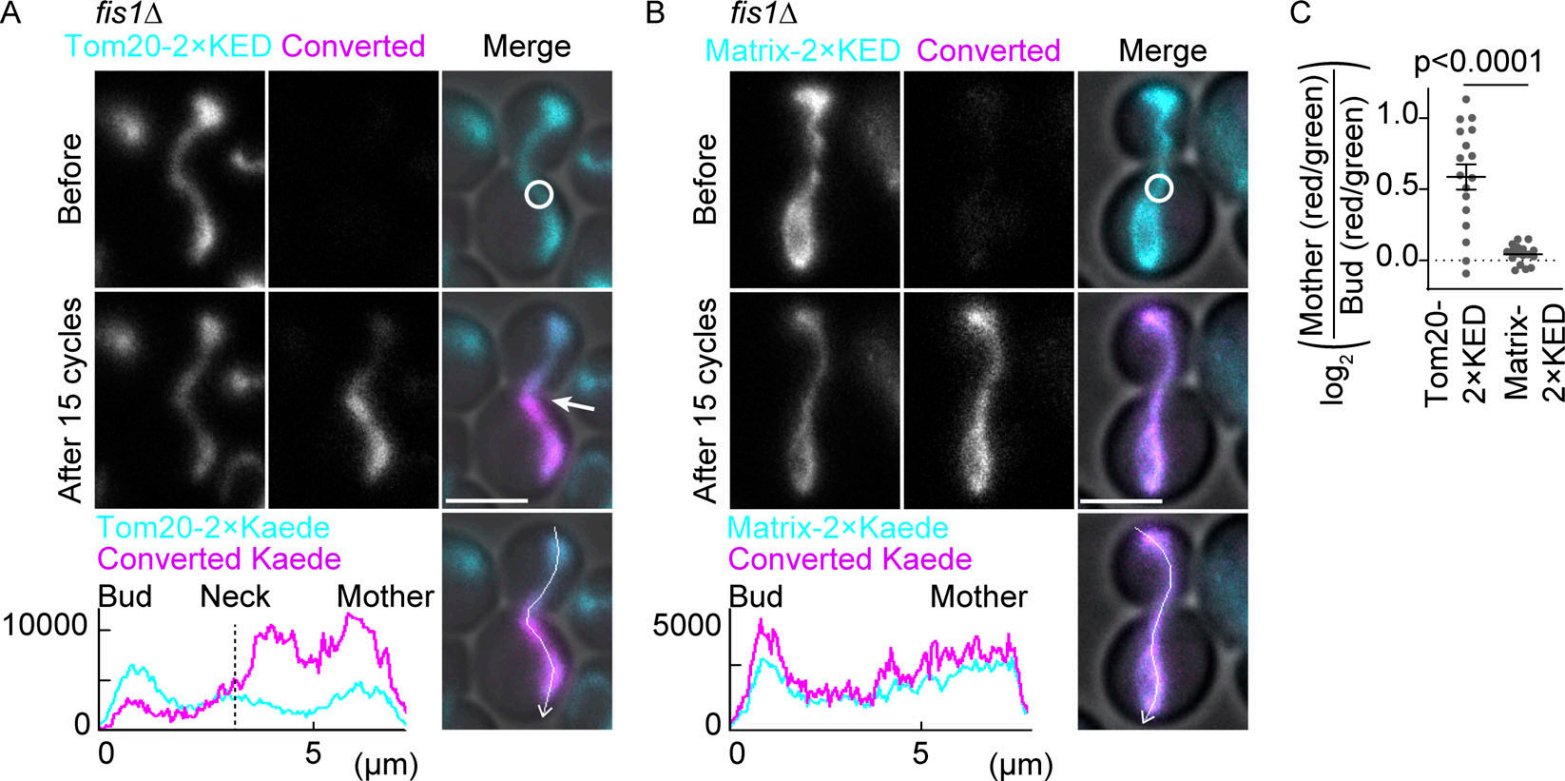
**Visualization of protein diffusion in mitochondria. (A and B)** Representative images from photoconversion experiments in *fis1*∆ cells expressing Tom20-2×Kaede in the absence of matrix-targeted fluorescence proteins (A) and *fis1*∆ cells expressing matrix-2×Kaede (B). **(C)** Quantification of converted-to-nonconverted Kaede fluorescence ratios (red/green) in the mother compartment compared with the bud compartment using Tom20-2×Kaede (*n* = 17 cells) or matrix-2×Kaede (*n* = 17 cells). Photoconversion was applied as indicated with white circles with 15 imaging-photoconversion cycles (10 s/cycle). The line graphs are the intensity profiles along the lines in the respective images after photoconversion. Images are a sum projection of five z-stacks taken at 0.5 μm intervals. Scale bar: 3 μm. Error bar: mean ± SE. Welch’s two-tailed *t* test was applied to compare the conversion ratios between two groups.

To test this notion more thoroughly, we considered whether the observed compartmentalization was due to mitochondrial fission events taking place after the continuity indicator, matrix-mCherry, had disappeared. Thus, we analyzed the diffusion of Tom20-GFP in mitochondrial fission-deficient *fis1*∆ and *dnm1*∆ mutant cells, where the mitochondrion remains a single continuous entity throughout the cell cycle until cytokinesis (Lackner and Nunnari, 2009; Otsuga et al., 1998; Bleazard et al., 1999; Mozdy et al., 2000). In these mutant cells, loss of Tom20-GFP fluorescence in the bud remained significantly delayed upon photobleaching in the mother cells, whereas Hem1-GFP was lost quickly both from the mother and bud, comparable with what was observed in wild-type cells (Fig. 3, A–C and Fig. S1, D–F). Similar results were obtained with the IMM reporter protein Atp1-GFP in *fis1*∆ cells (Fig. 3 D and Fig. S1 G). In all these cases, the bud neck appeared to form a boundary for fluorescence exchange; it was often observed that upon photobleaching in the mother cell, the fluorescence of the mitochondrion in the bud remained fairly continuous up until the bud neck and dropped right past it in the mother cell (Fig. 3 D, arrow). Moreover, a photoconversion experiment using Tom20-2×Kaede in *fis1*∆ cells demonstrated that the diffusion of photoconverted (red) Kaede was restricted at the bud neck, and the ratio of photoconverted and unconverted (green) Kaede changed abruptly at the bud neck upon repeated photoconversion in the mother, whereas soluble matrix 2×Kaede did not show any boundary (Fig. 4, A–C). Together, our data indicated that the exchange of mitochondrial membrane proteins between mother and bud was restricted, independent of mitochondrial fission. Furthermore, our data establish that compartmentalization of the mitochondrial membranes does not rely on the fission machinery. Accordingly, the studies hereafter were carried out in *fis1*∆ mutant cells to avoid unnecessary complications caused by mitochondrial fission.

### Mitochondria assemble diffusion barriers in their membranes at the bud neck

**Figure 5.**
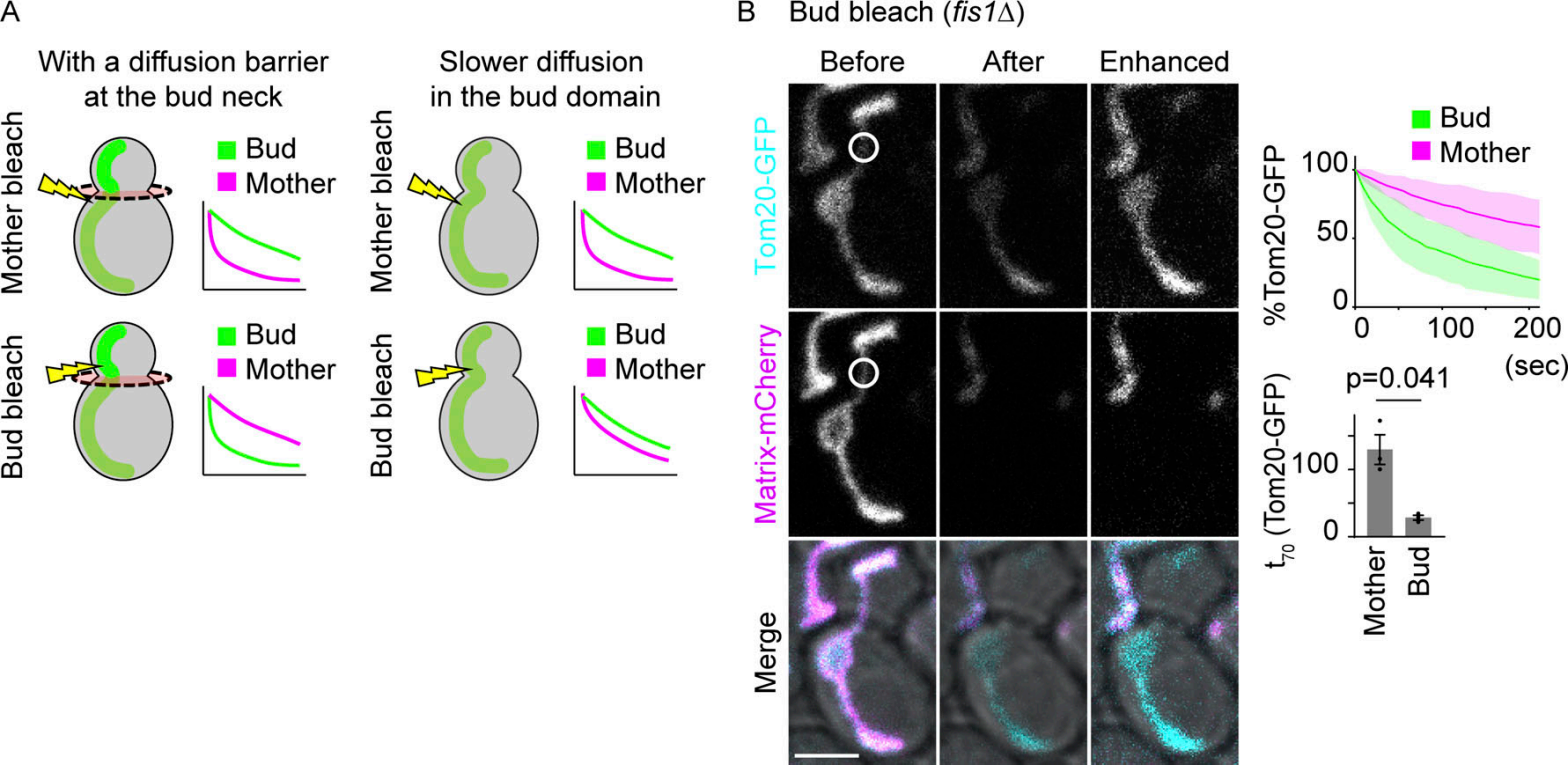
**A mitochondrial diffusion barrier exists at the bud neck. (A)** Two possible scenarios upon change of photobleaching areas from the mother (upper images) to bud (lower images). In the presence of a diffusion barrier at the bud neck, indicated by the red disk, bleaching in the bud would result in reversed bleaching curves (left). Slower diffusion in the bud compartment (absence of a diffusion barrier) would result in both compartments losing fluorescence in a similar manner upon bleaching in the bud (right). **(B)** Example images, pooled quantification of GFP FLIP, and t_70_ of GFP FLIP in *fis1*∆ cells expressing Tom20-GFP and matrix-mCherry (*n* = 34 cells, *n* ≥ 10 cells for each experiment). Photobleach was applied in the GFP and mCherry channels in the bud as indicated by white circles. Images are a sum projection of five z-stacks taken at 0.5-μm intervals. Scale bar: 3 μm. Shadows represent mean ± SD. Error bar: mean ± SE. Welch’s two-tailed *t* test was applied to compare the t_X_ in the mother and bud.

Delay of fluorescence loss in the bud upon photobleaching in the mother implies that a structure that restricts diffusion of membrane proteins, or a lateral diffusion barrier, might form in the mitochondrial membranes at the bud neck, reminiscent of the diffusion barriers observed in the ER and outer nuclear envelope membranes (Luedeke et al., 2005; Shcheprova et al., 2008). Alternatively, it could also mean that membrane proteins diffuse much slower in the bud than in the mother part of the mitochondria. To clarify what restricts the exchange of mitochondrial membrane proteins between the mother and bud, the photobleaching area was changed from the mother to the bud (Fig. 5 A). Under this new setup, a mitochondrial diffusion barrier at the bud neck should delay bleaching in the mother compartment compared with the bud, resulting in reversed bleaching curves (Fig. 5 A, left). If instead, the membrane marker were to be less mobile in the bud, the mother and bud curves should both decay slowly, without showing much kinetic difference (Fig. 5 A, right). Supporting the barrier model, photobleaching in the bud resulted in rapid fluorescence loss in the entire bud domain and delayed bleaching in the mother, reversing the curves observed when photobleaching was applied in the mother cell (Fig. 5 B). Thus, we concluded that lateral diffusion barriers form in the mitochondrial membranes at the bud neck.

### Mitochondria tethered to cell poles are compartmentalized

**Figure 6.**
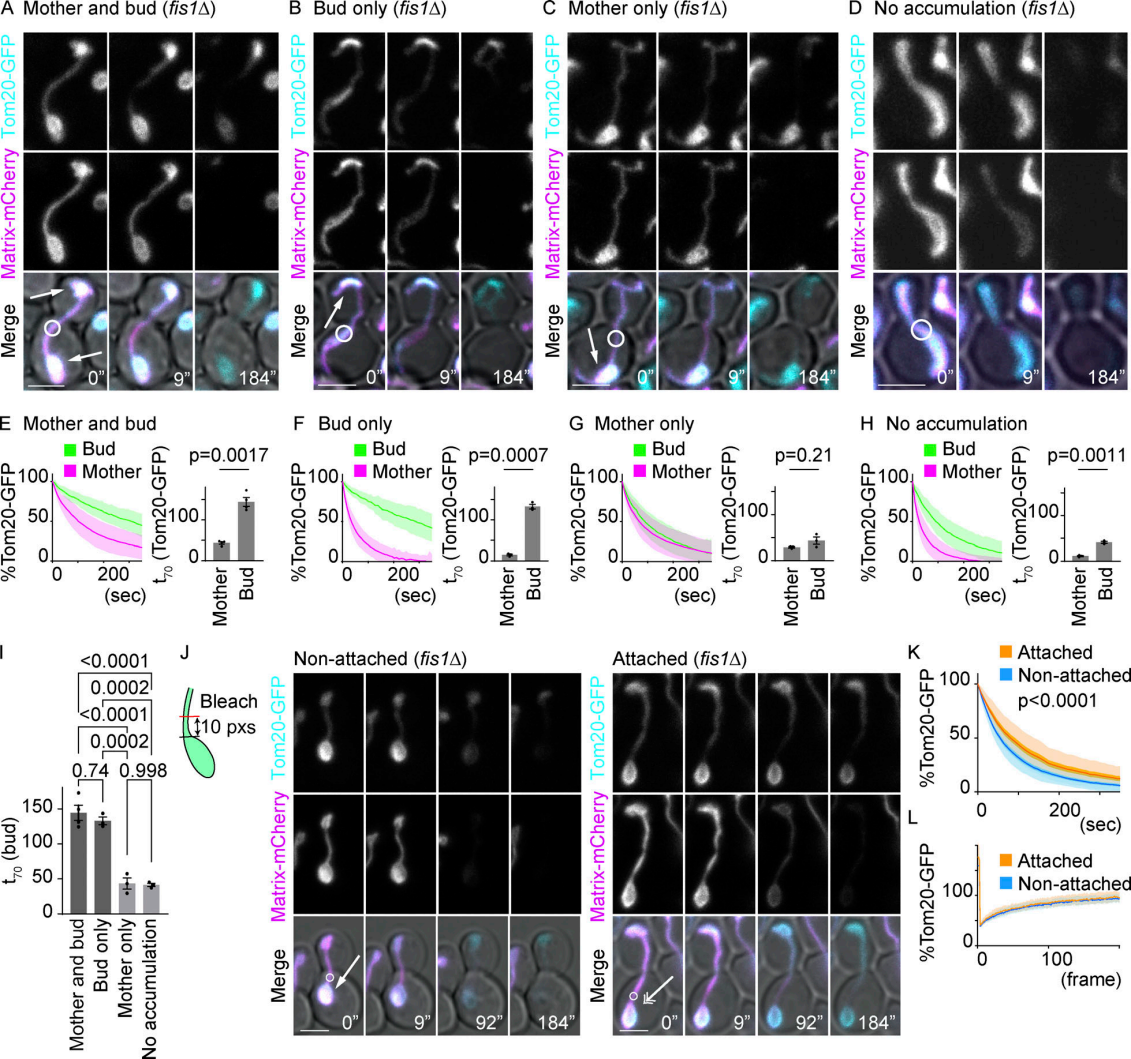
**Mitochondrial masses tethered at cell poles are compartmentalized. (A–H)** Dual-color FLIP in *fis1*∆ cells expressing GFP-tagged mitochondrial proteins and matrix-mCherry, categorized according to their morphologies. Example images (A–D), pooled quantification of GFP FLIP, and t_70_ of GFP FLIP from three or four independent experiments (E–H). Shadows represent mean ± SD. **(A and E)** Cells with mitochondrial accumulation both at the mother and bud poles (E; *n* = 40 cells, *n* = 10 cells for each experiment). **(B and F)** Cells with mitochondrial accumulation only at the bud pole (F; *n* = 28 cells, *n* ≥ 9 cells for each experiment). **(C and G)** Cells with mitochondrial accumulation only at the mother pole (G; *n* = 39 cells, *n* ≥ 10 cells for each experiment). **(D and H)** Cells without mitochondrial accumulation at the poles (H; *n* = 29 cells, *n* ≥ 9 cells for each experiment). Arrows indicate mitochondrial accumulation at cell poles. Welch’s two-tailed *t* test was applied to compare the t_X_ in the mother and bud. **(I)** t_70_ (bud) from E-H are compared among the morphology groups. t_70_ (bud) values in each category were compared by one-way ANOVA followed by Tukey’s method. **(J)** FLIP was applied in the mitochondrial tubules at 10 pxs away from the mitochondrial masses as illustrated in the model (left). Representative images of FLIP experiments in cells with mitochondrial masses without (left, indicated by an arrow) or with (right, indicated by a double arrow) attachment at the mother cell poles. **(K)** The pooled mother bleaching curves in cells that have mitochondrial masses with (*n* = 23 cells) or without (*n* = 24 cells) attachment at the mother cell poles. Light shadows represent mean ± SD and dark shadows represent mean ± SE. Data were fitted to the nonlinear regression curves and analyzed based on a null hypothesis “one curve for all data sets” and an alternative hypothesis of “different curve for each data set” using the extra sum-of-squares F-test. **(L)** FRAP was performed within the mitochondrial masses with (*n* = 35 cells) or without (*n* = 36 cells) attachment at the mother cell poles. Tom20-GFP fluorescence within the bleached area was obtained and normalized to the average of the final 200 data points (frame numbers 401–600; 100%). Shadows represent mean ± SD. Photobleaching was applied in GFP and mCherry channels as indicated with white circles. Images are a sum projection of five z-stacks taken at 0.5-μm intervals. Scale bar: 3 μm. Error bar: mean ± SE.

**Figure S2.**
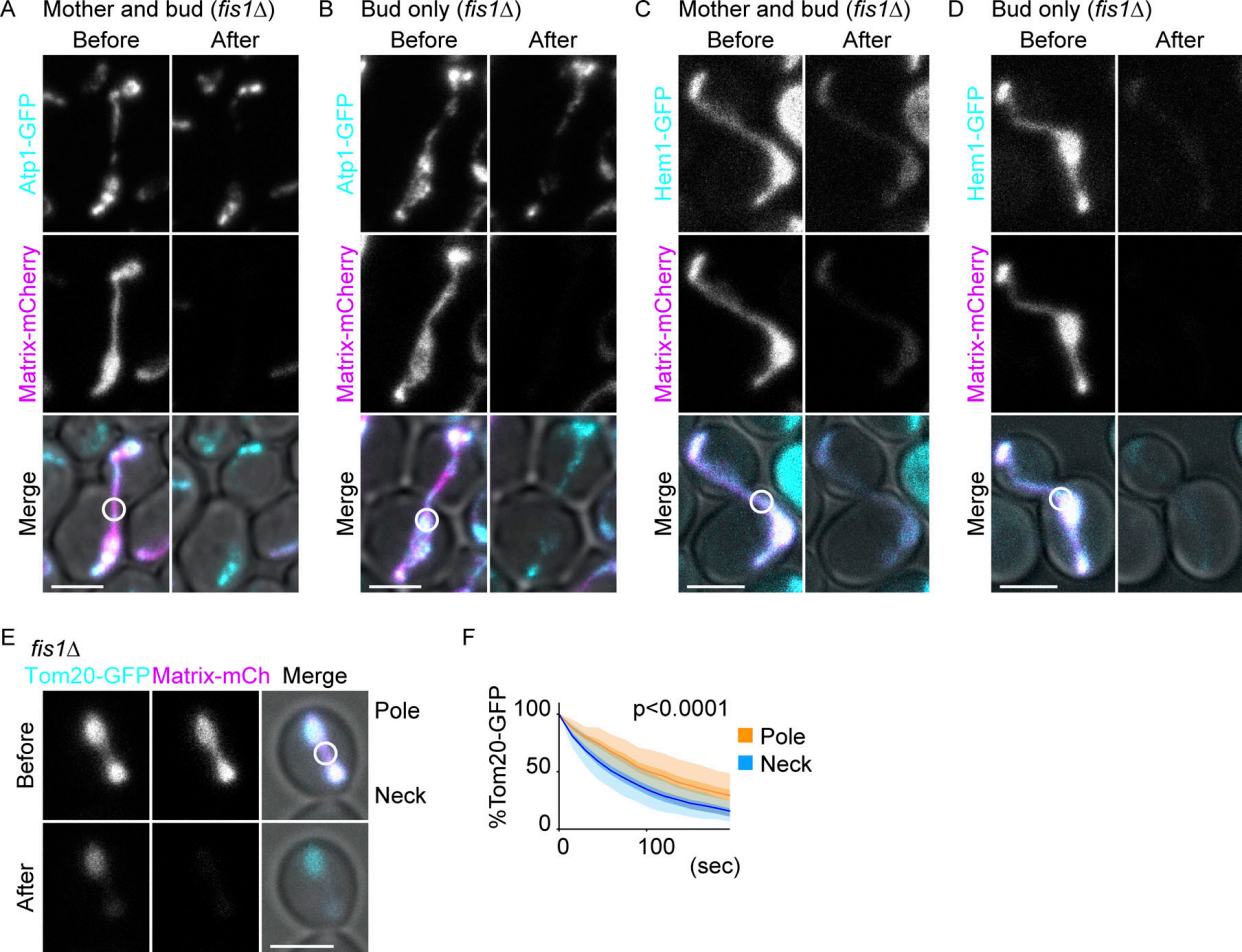
**Compartmentalization in mitochondria tethered to the cell poles. (A–F)** Dual-color FLIP in *fis1*∆ cells expressing GFP-tagged mitochondrial proteins and matrix-mCherry. **(A and B)** Representative images of *fis1*∆ cells expressing Atp1-GFP and matrix-targeted mCherry with mitochondrial accumulation both at the mother and bud poles (A) or only at the bud pole (B). Atp1-GFP shows delayed bleaching in the tethered mitochondrial masses whereas matrix-mCherry does not. **(C and D)** Representative images of *fis1*∆ cells expressing Hem1-GFP and matrix-targeted mCherry with mitochondrial accumulation both at the mother and bud poles (C) or only at the bud pole (D). Soluble matrix proteins do not show delayed bleaching in the tethered mitochondrial masses. **(E and F)** Representative images (E) and GFP quantification (F) of *fis1*∆ cells expressing Tom20-GFP and matrix-targeted mCherry after cytokinesis. Mitochondrial masses that are tethered to the former bud pole-side of daughter cells (Pole; *n* = 15 cells) show a delayed diffusion pattern of Tom20-GFP whereas those located at the former bud neck side (Neck; *n* = 15 cells) do not. Light shadows represent mean ± SD and dark shadows represent mean ± SE. Photobleach was applied in the GFP and mCherry channels as indicated by white circles. Images are a sum projection of five z-stacks taken at 0.5-μm intervals. Scale bar: 3 μm. Data were fitted to the nonlinear regression curves and analyzed based on a null hypothesis “one curve for all data sets” and an alternative hypothesis of “different curve for each data set” using the extra sum-of-squares F-test.

The Num1 protein tether mitochondria to the mother cell periphery (Lackner et al., 2013; Ping et al., 2016; Klecker et al., 2013), and Mfb1 and Mmr1 tether mitochondria to the mother and bud poles, respectively (Pernice et al., 2016; Swayne et al., 2011; Itoh et al., 2004; Frederick et al., 2008; Yang et al., 2022). Consistent with previous reports, we observed that mitochondria often accumulated at the mother and/or bud poles both in wild-type and *fis1*∆ mutant cells (Fig. 2 B and Fig. 3 A; and Fig. 6, A–D). In these cells, we noticed that the speed of fluorescence loss was greatly influenced by mitochondrial morphology. The decline of GFP fluorescence in the bud was much slower in cells with mitochondrial accumulation at the bud pole (Fig. 6, A, B, E, and F) compared with those without accumulation (Fig. 6, C, D, G–I). Similarly, fluorescence loss was slower in the mother when mitochondria accumulated at the mother pole (Fig. 6, A, C, E, and G) compared with those without (Fig. 6, B, D, F, and H). Similar changes in the FLIP patterns were observed for the IMM protein Atp1-GFP but not for the soluble matrix protein Hem1-GFP (Fig. S2, A–D). These data imply that there may be another mode of diffusion barrier in mitochondrial masses tethered to the cell poles.

To test whether pole-accumulated mitochondria are compartmentalized, we performed FLIP assays in *fis1∆* mutant cells with mitochondrial masses that are attached or not attached to the mother poles (Fig. 6 J). A small but significant delay in fluorescence loss was observed in attached mitochondrial masses compared with those without attachment to the mother tips, suggesting lateral compartmentalization of mitochondria attached to cell tips (Fig. 6, J and K). This was not caused by the membranes in attached mitochondrial masses being less fluid because fluorescence recovery after photobleaching (FRAP) rates were comparable between attached and non-attached masses (Fig. 6 L). This delayed diffusion in the tip-accumulated mitochondria seemed to remain after cytokinesis (in G1 cells) because a similar delay in diffusion was observed in the mitochondrial masses tethered on the former bud pole side compared with those on the former bud neck side (Fig. S2, E and F). These data imply that additional constraints separate tip-anchored mitochondrial masses from the rest of the network. Hereafter, we focused on the cells showing mitochondrial accumulation at both mother and bud tips because they represented the majority (57/102 cells) of big-budded *fis1*∆ mutant cells expressing matrix-mCherry.

### The IMM diffusion barrier is constitutive whereas that in the OMM forms under stress

Tom20 is a component of the translocase of the outer membrane (TOM) complex, which can be coupled to the translocase of the inner membrane (TIM) complex via protein import (Dekker et al., 1997); therefore, diffusion of Tom20 may be affected not only by the compartmentalization of the OMM but also by that of the IMM. Likewise, the mitochondrial F_1_F_o_ ATPase, which includes Atp1 as its subunit, is enriched in cristae rather than the inner boundary membrane (Busch, 2020). Indeed, the IMM protein, Atp1-GFP, localized to mitochondria in an inhomogeneous manner (Fig. 2 C). Therefore, loss of Atp1-GFP fluorescence by FLIP may reflect movements of cristae structures as well as actual diffusion of the protein. To overcome these problems and better characterize the mitochondrial diffusion barriers in each membrane, we changed the reporter proteins to Yta12-GFP (IMM) and Alo1-GFP (OMM). Yta12 is a mitochondrial inner membrane *m*-AAA protease that forms hetero-oligomers with Yta10 (Arlt et al., 1996). It preferentially localizes to the inner boundary membrane of IMM (Suppanz et al., 2009). Alo1 is a D-arabinono-1,4-lactone oxidase and is monomeric in the OMM (Huh et al., 1998; Burri et al., 2006; Zahedi et al., 2006).

**Figure 7.**
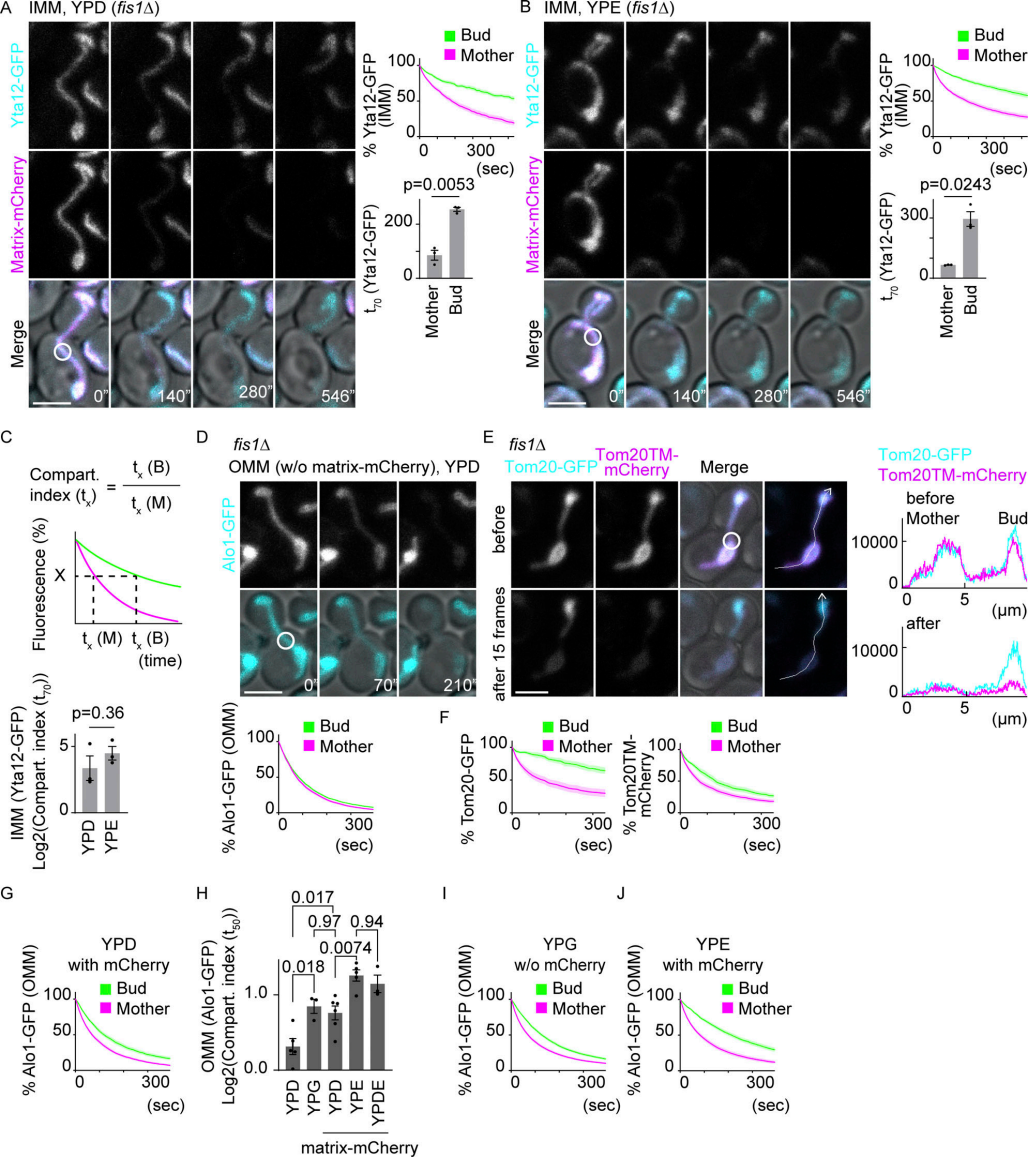
**IMM diffusion barriers exist constitutively and OMM diffusion barriers are formed in response to stresses. (A and B)** Dual-color FLIP in *fis1*∆ cells expressing Yta12-GFP (IMM protein) and matrix-mCherry. Representative images and pooled quantification of Yta12-GFP FLIP and t_70_ of Yta12-GFP are shown. Cells grown in YPD medium (A; *n* = 23 cells; *n* ≥ 6 cells for each experiment). Cells grown in YPE (B; *n* = 25 cells; *n* ≥ 5 cells for each experiment). **(C)** Compartmentalization indexes (t_X_) are calculated as t_X_ (time to reduce to X% of the total fluorescence) in the bud (t_X_ [B]) compared with t_X_ in the mother (t_X_ [M]) (upper panel). Compartmentalization indexes (t_70_) of Yta12-GFP FLIP data from A and B (lower panel). Welch’s two-tailed *t* test was applied to compare the t_X_ in the mother and bud. **(D)** GFP FLIP in *fis1*∆ cells expressing Alo1-GFP (OMM protein) in the absence of matrix-mCherry. Representative images and pooled quantification of Alo1-GFP FLIP are shown (*n* = 23 cells). **(E and F)** Dual-color FLIP in *fis1*∆ cells expressing Tom20-GFP and mCherry fused to the transmembrane region of Tom20 (Tom20TM-mCherry). Representative images and line graphs of the intensity profiles along the indicated lines (E). Quantification of Tom20-GFP and Tom20TM-mCherry (F; *n* = 17 cells). **(G)** Dual-color FLIP in *fis1*∆ cells expressing Alo1-GFP (OMM) and matrix-mCherry grown in YPD medium (*n* = 30 cells). **(H)** Compartmentalization indexes (t_50_) were calculated from Alo1-GFP (OMM protein) FLIP in *fis1*∆ cells grown on YPD, YPG, YPE, or YPDE in the presence or absence of matrix-mCherry. Data from at least three independent experiments are shown. *n* ≥ 5 cells for each clone, and a total of 42 cells (YPD without mCherry), 34 cells (YPG without mCherry), 54 cells (YPD with mCherry), 49 cells (YPE with mCherry), or 25 cells (YPDE with mCherry) were analyzed. Compartmentalization indexes (t_50_) were compared by one-way ANOVA followed by the Tukey’s method. **(I)** Single-color FLIP in *fis1*∆ cells expressing Alo1-GFP (OMM) in the absence of matrix-mCherry grown in YPG medium (*n* = 34 cells). **(J)** Dual-color FLIP in *fis1*∆ cells expressing Alo1-GFP (OMM) and matrix-mCherry grown in YPE medium (*n* = 49 cells). Photobleach was applied as indicated by white circles. Images are a sum projection of five z-stacks taken at 0.5-μm intervals. Scale bar: 3 μm. Shadows represent mean ± SE (hereafter, shadowed error bars in the FLIP experiments are changed to SE to compare the means among groups instead of showing distributions within a group). Error bar: mean ± SE.

**Figure S3.**
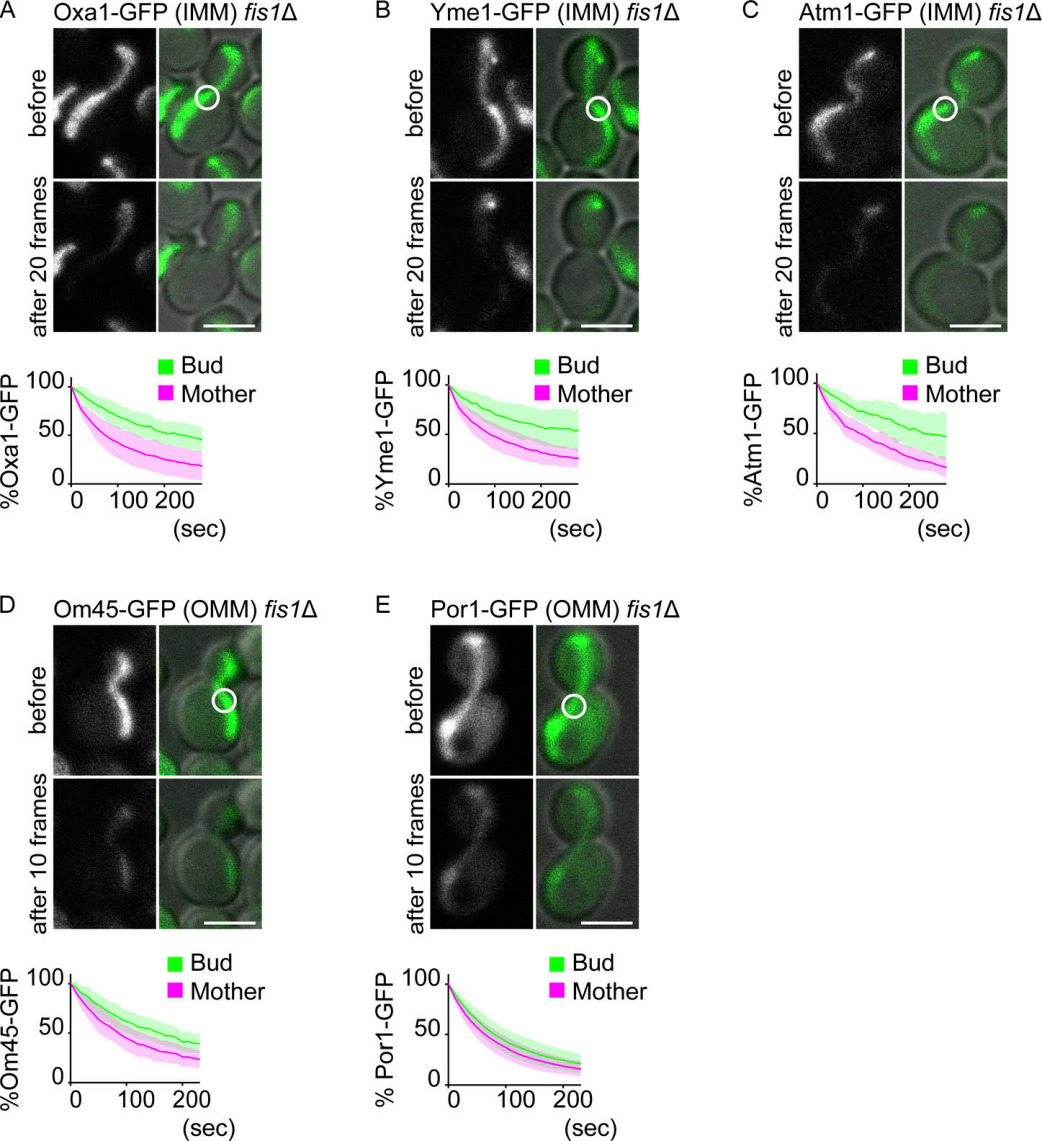
**Other IMM and OMM proteins. (A–E)** Single-color FLIP in *fis1*∆ cells expressing GFP-tagged mitochondrial IMM (A–C) or OMM (D and E) proteins in the absence of matrix-mCherry. Representative images and quantification of Oxa1-GFP (A; IMM, *n* = 10 cells), Yme1-GFP (B; IMM, *n* = 12 cells), Atm1-GFP (C; IMM, *n* = 10 cells), Om45-GFP (D; OMM, *n* = 12 cells), and Por1-GFP (E; OMM, *n* = 11 cells). Photobleach was applied in the GFP channel as indicated by white circles. Shadows represent mean ± SD. Images are a sum projection of five z-stacks taken at 0.5-μm intervals. Scale bar: 3 μm.

Diffusion of Yta12-GFP was analyzed using the FLIP assay as above (Fig. 3). Similarly to Tom20-GFP and Atp1-GFP, Yta12-GFP fluorescence decay was delayed in the bud of cells grown in a rich medium (YPD), confirming the existence of a lateral diffusion barrier in the IMM at the bud neck, independent of cristae localization (Fig. 7 A). Similar patterns with the diffusion restriction at the bud neck were observed for other IMM proteins, Oxa1-GFP, Yme1-GFP, and Atm1-GFP, confirming the existence of a diffusion barrier in the IMM (Fig. S3, A–C). Growing the cells in a non-fermentable medium with ethanol as a sole carbon source (YPE) slightly decreased the bleaching speed of Yta12-GFP, both in the mother and bud (Fig. 7 B), although no big difference was observed in the “compartmentalization index” (t_X_; Boettcher et al., 2012), defined here as t_X_ (time to reduce to X% of the total fluorescence) in the bud compared with t_X_ in the mother (compartmentalization index $\left(t_{X}\right)=t_{X}\left(bud\right)/t_{X}\left(mother\right)$; Fig. 7 C). These data suggest that the IMM diffusion barrier is constitutive and not noticeably regulated by growth conditions.

Strikingly, the diffusion of Alo1-GFP showed a distinct pattern. When the cells were grown in the YPD medium, the Alo1-GFP fluorescence decayed as quickly in the bud as in the mother cell (Fig. 7 D). Other well-known OMM proteins, OM45-GFP and Por1-GFP, also showed the same diffusion pattern, suggesting that there is indeed no diffusion barrier in the OMM of cells grown in a rich medium, validating Alo1-GFP as a legitimate OMM protein marker (Fig. S3, D and E). To better understand the diffusion pattern observed with Tom20, we characterized the diffusion of Tom20TM-mCherry, where mCherry was fused to the transmembrane region of Tom20, thus preventing interaction with the TOM complex. Strikingly, Tom20TM-mCherry was lost quickly both from the mother and bud similarly to the other OMM proteins tested above, whereas Tom20-GFP expressed in the same cells showed delayed diffusion in the bud upon photobleaching in the mother (Fig. 7, E and F). These data confirm that no lateral diffusion barrier assembles in the OMM under optimal growth conditions and that the compartmentalization observed for Tom20-GFP under such conditions must have indeed depended on the interaction of the reporter protein with other TOM complex and most probably with the TIM complex in the IMM. Therefore, we used Alo1-GFP as an OMM protein marker hereafter. Interestingly, expression of matrix-mCherry tended to introduce a moderate delay between mother and bud (Fig. 7, G and H), suggesting that it slightly induces the formation of a barrier in the OMM. Supporting the notion of an inducible barrier in the OMM, growth on glycerol as a non-fermentable carbon source (YPG) induced the same moderate compartmentalization of the OMM (Fig. 7, H and I). This delay was enhanced in cells grown on ethanol as a carbon source (YPE), resulting in a significantly higher compartmentalization index (t_50_; Fig. 7, H and J). Furthermore, the addition of 2% ethanol to the glucose-based medium (YPDE) was sufficient to induce the OMM diffusion barrier to a comparable level to that observed in the YPE medium (Fig. 7 H). In YPDE, ethanol is not used as a carbon source, and the metabolism is driven essentially by the fermentation of glucose. These data suggest that the OMM assembles a diffusion barrier only and specifically under defined conditions, which are not specifically associated with respiration but more likely with stresses, such as ethanol or the overexpression of mCherry (Kintaka et al., 2016) in the mitochondrion.

### IMM diffusion barrier at the bud neck is partially dependent on Shs1 and Bud6

**Figure 8.**
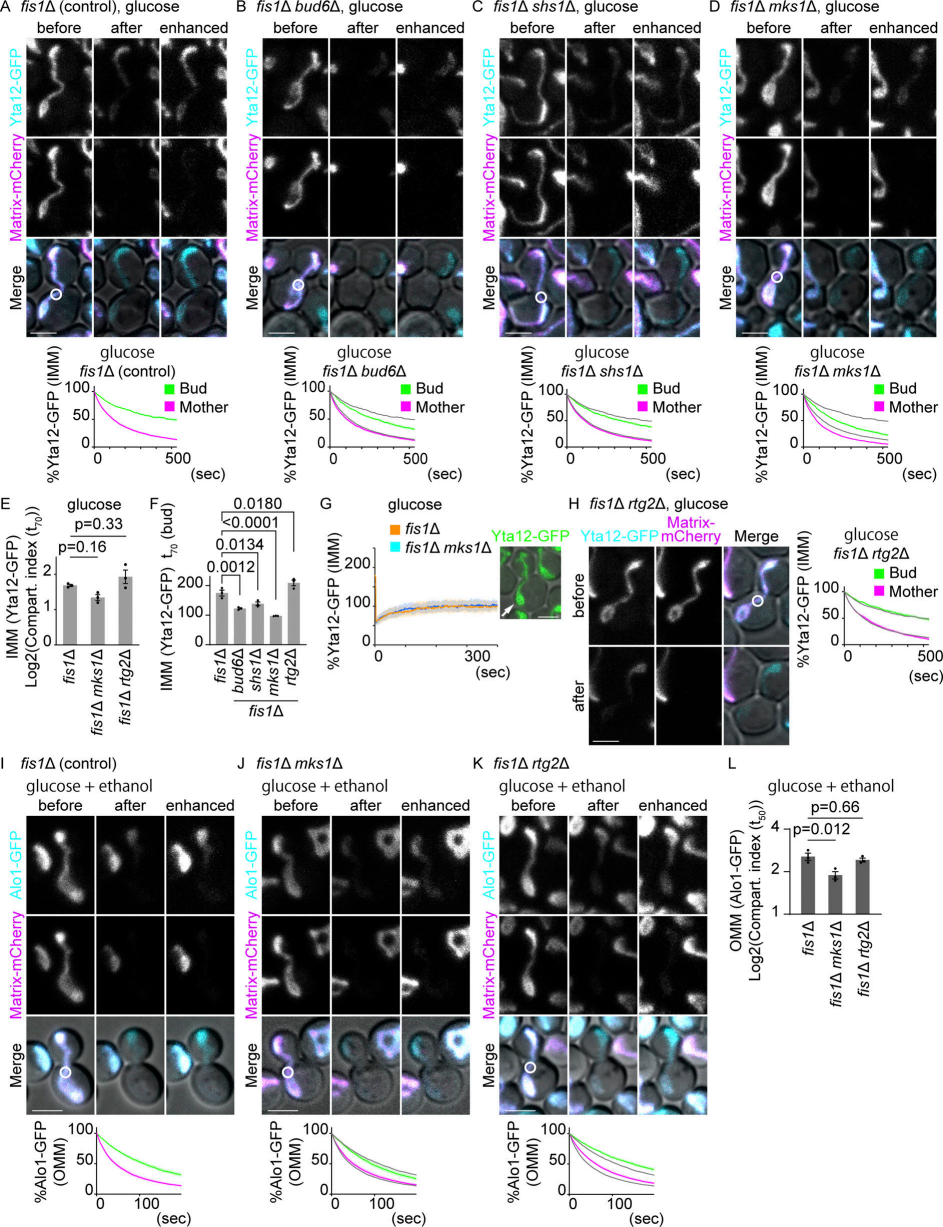
**Regulation of IMM and OMM diffusion barriers. (A–D)** Dual-color FLIP cells expressing Yta12-GFP (IMM) and matrix-mCherry. Representative images and quantification of Yta12-GFP FLIP in *fis1*∆ (A; *n* = 37 cells), *fis1*∆ *bud6*∆ (B; *n* = 34 cells), *fis1*∆ *shs1*∆ (C; *n* = 35 cells), and *fis1*∆ *mks1*∆ (D; *n* = 36 cells) cells. Yta12-GFP bleaching curves in *fis1*∆ cells are overlayed as gray lines (the same set of *fis1*∆ data from A are overlayed in B–D and H). **(E)** Compartmentalization indexes (t_70_) were calculated from three independent clones (the same set of data as D and H). *n* ≥ 10 cells for each experiment. Shadows represent mean ± SE. **(F)** t_70_ values for the bud curves were calculated from three independent experiments using the same set of data as in A–E and H. **(G)** FRAP was performed within the mitochondrial masses that are attached at mother cell poles as exemplified (right) in *fis1*∆ cells (*n* = 45 cells) or in *fis1*∆ *mks1*∆ cells (*n* = 45 cells). Yta12-GFP fluorescence within the bleached area was obtained and normalized to the average of the final 200 data points (frame numbers 401–600; 100%). Shadows represent mean ± SD. **(H)** Representative images and quantification of Yta12-GFP FLIP in the presence of matrix-mCherry in *fis1*∆ *rtg2*∆ cells (*n* = 33 cells). Bleaching curves of *fis1*∆ cells are overlayed as in B–D. **(I–K)** Dual-color FLIP in cells expressing Alo1-GFP (OMM) and matrix-mCherry grown on agar plates containing SD + 2% glucose and 2% ethanol. Representative images and quantification of Alo1-GFP FLIP in *fis1*∆ (*n* = 30 cells), *fis1*∆ *rtg2*∆ (*n* = 30 cells), and *fis1*∆ *mks1*∆ cells (*n* = 30 cells). Alo1-GFP bleaching curves in *fis1*∆ cells are overlayed as gray lines (the same set of *fis1*∆ data from I are overlayed in J and K). Shadows represent mean ± SE. **(L)** Compartmentalization indexes (t_50_) were calculated from three independent clones with 10 cells per clone (the same data as I–K). Error bar: mean ± SE. Photobleach was applied in the GFP and mCherry channels as indicated by white circles. Images are a sum projection of five z-stacks taken at 0.5 μm intervals. Scale bar: 3 μm. Statistical analyses were performed by one-way ANOVA followed by Dunnett’s method.

**Figure S4.**
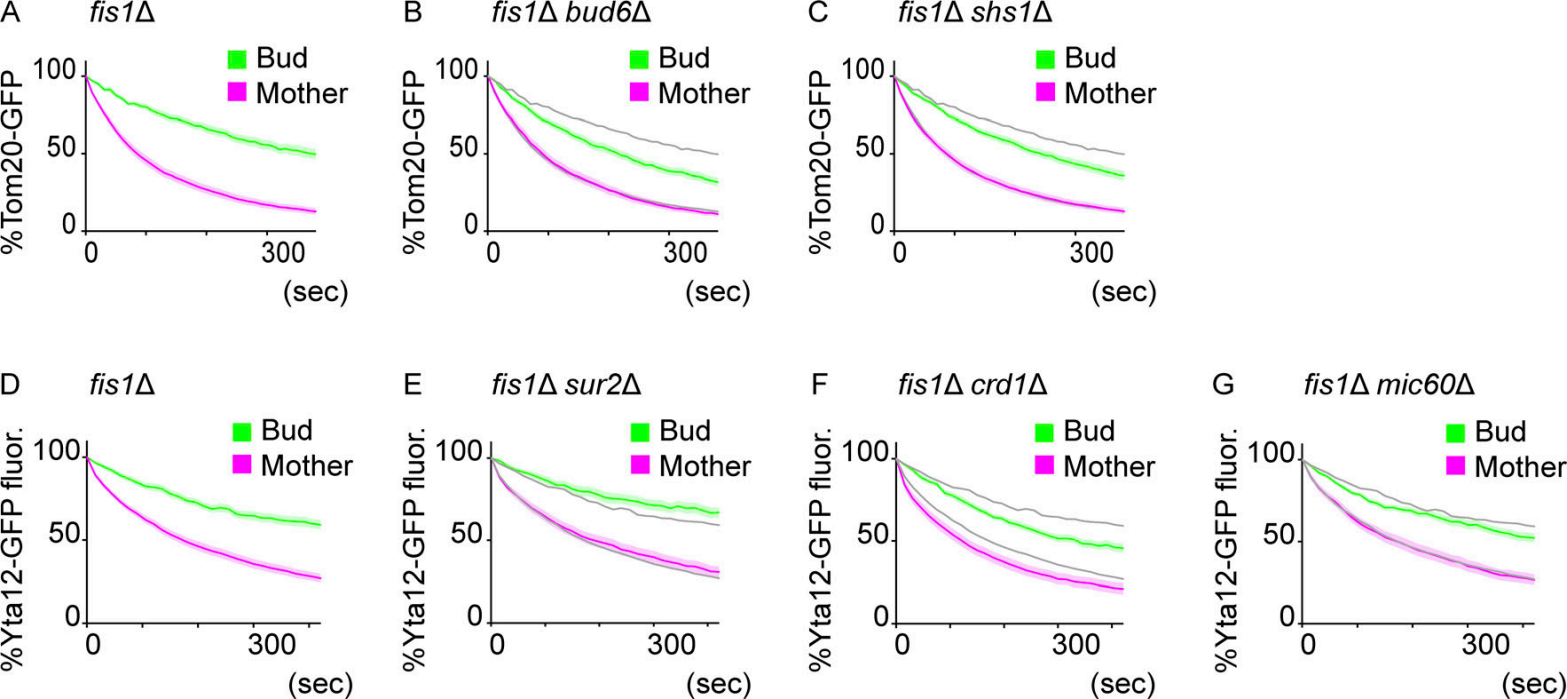
**Regulation of IMM diffusion barriers. (A–C)** Dual-color FLIP in *fis1*∆ cells expressing Tom20-GFP and matrix-mCherry. Quantification of Tom20-GFP FLIP in *fis*1∆ (A; *n* = 30 cells), *fis*1∆ *bud6*∆ (B; *n* = 23 cells), *fis1*∆ *shs1*∆ (C; *n* = 30 cells) cells. Tom20-GFP bleaching curves from A are overlayed in B and C as gray lines. **(D–G)** Dual-color FLIP in *fis1*∆ cells expressing Yta12-GFP and matrix-mCherry. Quantification of Yta12-GFP FLIP in *fis*1∆ (D; *n* = 36 cells), *fis1*∆ *sur2*∆ (E; *n* = 21 cells), *fis*1∆ *crd1*∆ (F; *n* = 20 cells), and *fis*1∆ *mic60*∆ (G; *n* = 25 cells). Yta12-GFP bleaching curves from D are overlayed in E–G as gray lines. Shadows represent mean ± SE.

Several bud neck proteins, including septins and Bud6, are required for establishing the diffusion barriers on the ER and outer nuclear envelope membranes (Shcheprova et al., 2008; Luedeke et al., 2005). Septins are membrane-interacting cytoskeletal GTPases that form rings at the bud neck and participate in cell polarization and cytokinesis (Caudron and Barral, 2009; Spiliotis and McMurray, 2020). Bud6 is a polarisome component that localizes first at the budding site and later at the bud neck (Casamayor and Snyder, 2002; Sheu et al., 2000). We asked whether they also played roles in establishing the IMM diffusion barriers. The delay of Yta12-GFP fluorescence loss in the bud was reduced, but not abrogated, in *bud6*∆ *fis1*∆ double mutant cells compared with *fis1*∆ control cells (Fig. 8, A and B). An even smaller reduction of barrier strength was observed by the deletion of the non-essential septin, Shs1 (Fig. 8 C). In both cases, the mother bleaching curves almost completely overlapped with that in *fis1*∆ cells, indicating that the speed of protein diffusion or the diffusion barrier at the pole-anchored mitochondria was not detectably affected. Similar reductions of the barrier strength in Bud6- or Shs1-deficient cells were observed using Tom20-GFP as a reporter (Fig. S4, A–C). These data indicate that Shs1 and Bud6 affect the IMM diffusion barrier at the bud neck, but to a much milder extent than observed for the barriers located in the ER membrane and the outer nuclear envelope (Clay et al., 2014; Luedeke et al., 2005). Alternatively, loss of polarity may affect the morphology and organization of mitochondria as reported recently (Yang et al., 2022), which in turn may affect the diffusion patterns. These data suggest that Shs1 and Bud6 are partially but not absolutely required for the formation of the IMM diffusion barrier at the bud neck. The lateral compartmentalization of the mitochondrial membrane might follow in part similar spatial cues as those of the ER and nuclear diffusion barriers.

Diffusion barriers in the ER and outer nuclear envelope are composed of thicker lipid bilayers dependent on ceramide (Prasad et al., 2020). In mitochondria, however, the loss of Sur2, which is required for phytoceramide synthesis and thus for ER and outer nuclear membrane diffusion barriers, did not weaken the IMM diffusion barrier (Fig. S4, D and E). Instead, the loss of cardiolipin, an IMM-specific phospholipid, caused a partial reduction of the IMM diffusion barrier (Fig S4 F). In contrast, the loss of a mitochondrial contact site and the cristae organizing system (MICOS) complex component, Mic60 (Rabl et al., 2009), did not have a large impact on the diffusion pattern of Yta12-GFP (Fig. S4 G), suggesting that the changes observed in the *crd1*Δ cells are not simply due to the altered cristae structure. These data suggest that the mitochondrial barrier involves distinct molecular mechanisms than the ER diffusion barrier, which is composed of the local accumulation of ceramide in the membrane bilayer at the site of the barrier.

### Mitochondrial retrograde signaling negatively regulates the mitochondrial diffusion barriers

Mitochondrial DNA encodes only a subset of oxidative phosphorylation complexes (Taanman, 1999). Thus, any mitochondrial or non-mitochondrial proteins that are involved in the regulation of mitochondrial diffusion barriers are expected to be encoded in the nuclear genome. The fact that the strength of mitochondrial diffusion barriers can be regulated implies the existence of a feedback mechanism. Therefore, we tested the involvement of the pathway that signals from mitochondria to the nucleus, the mitochondrial retrograde (RTG) pathway (Butow and Avadhani, 2004). Mks1 is a negative regulator of the RTG pathway, and Rtg2 acts as an inhibitor of Mks1 and thereby activates the pathway. Remarkably, Yta12-GFP fluorescence decay was accelerated in the mother and even more severely in the bud of the *mks1*∆ *fis1*∆ double-mutant cells (Fig. 8 D). Although the compartmentalization index (t_70_) did not show a statistically significant reduction in *mks1*∆ *fis1*∆ double-mutant cells, likely due to the changes both in the mother and bud curves, t_70_(bud) showed a clear reduction (Fig. 8, E and F). This did not appear to be due to increased overall membrane fluidity because FRAP experiments within mitochondrial masses showed comparable recovery speeds between *fis1*∆ and *mks1*∆ *fis1*∆ cells (Fig. 8 G). Therefore, quicker bleaching in the mother and bud likely reflected the reduction of lateral compartmentalization both in the tethered mitochondria and at the bud neck. On the other hand, bleaching curves were not largely affected in *rtg2*∆ *fis1*∆ cells, with a very modest increase of t_70_ (bud), suggesting that the RTG pathway is likely at the resting state under optimal culture conditions (Fig. 8, E, F, and H). Likewise, the loss of Mks1, but not Rtg2, caused a reduction of the OMM diffusion barrier under the OMM barrier-inducing condition (YPDE; Fig. 8, I–L). These data indicate that activation of mitochondrial retrograde signaling represses the compartmentalization of IMM, both at the bud neck and in tethered mitochondria, and of the OMM of stressed cells.

## Discussion

In this study, we show that mitochondria are laterally compartmentalized independently of physical separation. Our data indicate that this compartmentalization relies on the formation of diffusion barriers that are positively regulated by spatial cues and negatively regulated by retrograde signaling. The IMM diffusion barrier is constitutive at the bud neck and at tip-anchored mitochondrial masses. The OMM barrier forms in response to stresses such as ethanol stress and overexpression of mCherry in the matrix. These data imply that spatial cues from outside of mitochondria have to reach IMM even in the absence of the OMM diffusion barrier. Indeed, loss of the non-essential septin, Shs1, or the bud neck protein, Bud6, moderately reduces the IMM barrier strength, supporting the existence of yet unknown signaling from outside of mitochondria reaching IMM. The composition and molecular mechanisms of mitochondrial diffusion barrier formation will need further study.

Diffusion barriers in the ER and outer nuclear envelope of dividing *S. cerevisiae* mediate retention of partitioned materials, such as aging factors, in the mother cells to ensure the rejuvenation of their daughter cells (Shcheprova et al., 2008; Clay et al., 2014; Saarikangas et al., 2017). Currently, the physiological importance of mitochondrial diffusion barriers is unclear. By analogy with other diffusion barriers, we speculate that mitochondrial diffusion barriers may facilitate their quality control and partitioning of mitochondrial fitness. Regulated partitioning by diffusion barriers may drive the rejuvenation of mitochondria in the bud similar to the biased transport and tethering of fit mitochondria (Itoh et al., 2002, 2004; Altmann et al., 2008; Förtsch et al., 2011; McFaline-Figueroa et al., 2011; Swayne et al., 2011; Higuchi et al., 2013; Klecker et al., 2013; Lackner et al., 2013; Pernice et al., 2016; Ping et al., 2016). Interestingly, proteotoxic stress-induced cytosolic aggregates are associated with mitochondria and rarely pass through the bud neck as if they encounter an invisible barrier (Zhou et al., 2014). The stress-induced mitochondrial OMM diffusion barrier that we describe here may be involved in the retention of such cytosolic oligomers in the mother cell. Identifying specific factors that only affect the mitochondrial diffusion barriers will be required to test these ideas and study the physiological importance of mitochondrial compartmentalization.

## Materials and methods

### Yeast strains

Yeast strains used in this study are listed in Table S1. All yeast strains were constructed according to standard genetic techniques (Janke et al., 2004) and are isogenic to BY4741. Tom20-GFP, Atp1-GFP, Hem1-GFP, Yta12-GFP, Alo1-GFP, Oxa1-GFP, Yme1-GFP, Atm1-GFP, and Om45-GFP were obtained from Yeast GFP collection (Thermo Fisher Scientific). All cultures were grown at 30°C on YPD (yeast extract, peptone, and 2% glucose), YPE (yeast extract, peptone, and 2% ethanol), YPDE (yeast extract, peptone, 2% glucose, and 2% ethanol), or SD (synthetic defined with 2% glucose) agar plates, or in YPD, YPG, YPE, or SD liquid medium as indicated.

### Plasmids

pYX142-mtGFP was a gift from Dr. Benedikt Westermann (plasmid #45050; Addgene; https://n2t.net/addgene:45050; Westermann and Neupert, 2000) (Universität Bayreuth, Bayreuth, Germany). GFP sequence was exchanged to mCherry or 2×Kaede in pYX142-mtGFP to obtain a plasmid encoding matrix-targeted mCherry, pYB4017 su9-mCherry, or matrix-targeted 2×Kaede, pYB4019 su9-2×Kaede. The matrix-targeting signal of pYB4017 su9-mCherry was exchanged to the first 35 amino acids of *S. cerevisiae* Tom20 to obtain pYB4018 Tom20TM-mCherry. pFA6a 2×Kaede-kanMX6 was a gift from Dr. Hayashi Yamamoto (Yamamoto et al., 2012) (Nippon Medical School, Tokyo, Japan).

### Microscopy

All strains were grown at 30°C in SD-leucine (2% glucose) liquid medium unless otherwise stated. Cells were inoculated in SD-leucine liquid medium and diluted twice prior to analysis keeping OD_600_ always below 0.8 for at least 24 h and then used for analysis at OD_600_ 0.2–0.8. Cells were then harvested, immobilized on a 2% agar pad containing SD-leucine medium, and imaged at 30°C on Olympus Fluoview 3000 confocal microscope with a ×60/1.35 NA objective and GaAsP PMTs detector and captured with FluoView software (FV10-ASW; Olympus). Image analysis was performed using Fiji ImageJ (Schindelin et al., 2012).

### FLIP experiments

FLIP experiments were performed as previously described (Shcheprova et al., 2008). Briefly, photobleaching was applied on an ROI as indicated in the figures with a line scan of 5 pixels at 9 × software magnification. Imaging and photobleaching were repeated in areas, including the cell of interest and in at least three control cells. Five z-stacks at 0.5-μm intervals were taken for each time point.

FLIP quantification was performed as follows. Sum projection was applied for the five z-stacks taken at 0.5-μm intervals. ROIs were obtained by manually outlining the mother, bud, three neighboring cells, and background. The mean fluorescence signals were quantified using sum projection, and the values were set to 100% at the beginning of the experiments. Data from one clone were pooled to obtain t_X_ (bud) and t_X_ (mother), and data from at least three experiments were pooled to obtain a bleaching curve for the strain as indicated. The line graphs were generated using the Multichannel Plot Profile function of the BAR plugin (Tiago et al., 2015) after background subtraction with a rolling ball filter = 50.

### Photoconversion experiments

Cells were prepared and imaged as in FLIP experiments. Repeated photoconversion with C-terminally 2×Kaede-tagged Tom20 or matrix-targeted 2×Kaede was performed in ROIs as indicated in the figure with a line scan of five pixels at 9 × software magnification. After imaging of the green and red channels (“before”), the photoconversion using the 405 nm light at 0.1% intensity and imaging of the green channel were repeated 15 times (10 s/cycle), and the green and red channels were imaged again (“after 15 cycles”). Five z-stacks at 0.5-μm intervals were taken for each time point and sum projection was applied for the five z-stacks. The line graphs were generated using the Multichannel Plot Profile function of the BAR plugin (Tiago et al., 2015) after background subtraction with a rolling ball filter = 50.

### FRAP experiments

Cells were prepared and imaged as in FLIP experiments. For FRAP experiments, 5 × 5 pixels at 9 × software magnification were used for imaging and photobleaching. Three images were taken first followed by photobleaching once for 2.0 µs/pixel (total 7.73 ms/frame) at 100% laser power and subsequent imaging (free run) at 0.2% laser power. FRAP quantification was performed using Fiji ImageJ (Schindelin et al., 2012). The mean fluorescence signals from 5 × 5 pixels were obtained and the mean values of frame numbers 401–600 were set to 100% with the premise that there is no immobile fraction, judging from the FLIP experiments (the difference between the original and final fluorescence strengths is due to the bleached amount, which is not negligible compared with the total fluorescence). All experiments were performed with three independent clones.

### Statistical analysis

Data are presented as mean ± SD or ± SE as indicated. Statistical analysis was performed using GraphPad Prism 9.4.0. Data distribution was assumed to be normal, but this was not formally tested. Two groups of data were evaluated by two-tailed Welch’s *t* test. Multiple comparisons were performed by one-way ANOVA followed by Tukey’s method for Figs. 6 and 7 (comparison of all) or Dunnett’s method for Fig. 8 (comparison of multiple test groups with one control group). In Fig. 6 K and Fig. S2 F, *P*-values were calculated as follows: data were fitted to the nonlinear regression curves and analyzed based on a null hypothesis “one curve for all data sets” and an alternative hypothesis of “different curve for each data set” using the extra sum-of-squares F-test.

### Online supplemental material

Fig. S1 shows the quantification of matrix-mCherry FLIP related to Figs. 2 and 3. Fig. S2 shows examples of the protein diffusion patterns in the tethered mitochondria related to Fig. 6. Fig. S3 shows the FLIP data of other OMM and IMM marker proteins related to Fig. 7. Fig. S4 shows the FLIP data of factors that affect the IMM barrier strength related to Fig. 8. Table S1 shows yeast strains generated and used in this study.

## Supplementary Material

Table S1 shows yeast strains generated and used in this study.

## Data Availability

Data are available in the article itself and its supplementary materials. Original data, strains, and plasmids generated in this study are available from the corresponding author upon request.
