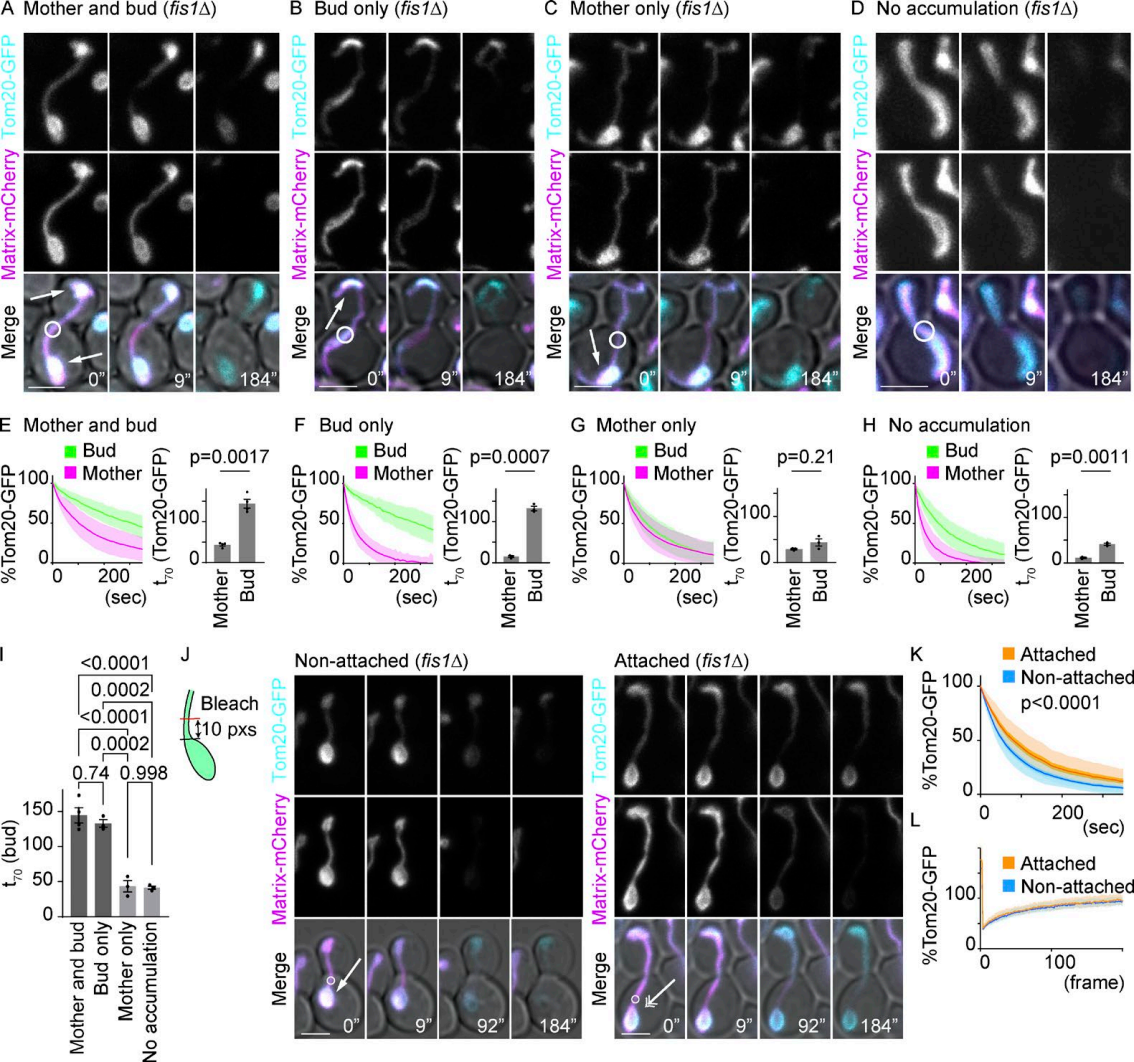# Correction: Fission-independent compartmentalization of mitochondria during budding yeast cell division

**DOI:** 10.1083/jcb.20221104803112024c

**Published:** 2024-03-15

**Authors:** Saori R. Yoshii, Yves Barral

Vol. 223, No. 3 | https://doi.org/10.1083/jcb.202211048 | January 5, 2024

After publication, the authors discovered that in Figure 6 A, the 9'' and 184'' matrix-mCherry images were mistakenly swapped. The corrected Figure 6 is shown here. The figure legend remains unchanged.

The conclusions of the paper are not affected by this error, and all discussion of the data presented remains correct.

This error appears in the print and any PDF downloaded prior to March 13, 2024. The authors apologize for any confusion this may have caused.

**Figure:**