# Supplementary material for: Fission-independent compartmentalization of mitochondria during budding yeast cell division
Source: J Cell Biol. 2024 Jan 5;223(3):e202211048. doi: 10.1083/jcb.202211048 (PMC10783438; doi:10.1083/jcb.202211048)
Supplement: Table S1 — shows yeast strains generated and used in this study. [file JCB_202211048_TableS1.docx]

Table S1. Yeast strains generated and used in this study

| Mating type | Genotype |
| --- | --- |
| a | Tom20-GFP:His3MX6; pYB4017 su9-mCherry |
| a | Tom20-GFP:His3MX6 fis1::HphNT1; pYB4017 su9-mCherry |
| a | Tom20-GFP:His3MX6 dnm1::HphNT1; pYB4017 su9-mCherry |
| a | Tom20-GFP:His3MX6 fis1::HphNT1 bud6::NatNT2; pYB4017 su9-mCherry |
| a | Tom20-GFP:His3MX6 fis1::HphNT1 shs1::NatNT2; pYB4017 su9-mCherry |
| a | Tom20-2xKaede:KanMX6 fis1::HphNT1 |
| a | Tom20-GFP:His3MX6 fis1::HphNT1; pYB4018 Tom20TM-mCherry |
| a | Hem1-GFP:His3MX6; pYB4017 su9-mCherry |
| a | Hem1-GFP:His3MX6 fis1::HphNT1; pYB4017 su9-mCherry |
| a | Atp1-GFP:His3MX6; pYB4017 su9-mCherry |
| a | Atp1-GFP:His3MX6 fis1::HphNT1; pYB4017 su9-mCherry |
| a | Yta12-GFP:His3MX6 fis1::HphNT1; pYB4017 su9-mCherry |
| a | Yta12-GFP:His3MX6 fis1::HphNT1 bud6::NatNT2; pYB4017 su9-mCherry |
| a | Yta12-GFP:His3MX6 fis1::HphNT1 shs1::NatNT2; pYB4017 su9-mCherry |
| a | Yta12-GFP:His3MX6 fis1::HphNT1 mks1::NatNT2; pYB4017 su9-mCherry |
| a | Yta12-GFP:His3MX6 fis1::HphNT1 rtg2::NatNT2; pYB4017 su9-mCherry |
| a | Yta12-GFP:His3MX6 fis1::HphNT1 sur2::NatNT2; pYB4017 su9-mCherry |
| a | Yta12-GFP:His3MX6 fis1::HphNT1 crd1::NatNT2; pYB4017 su9-mCherry |
| a | Yta12-GFP:His3MX6 fis1::HphNT1 mic60::NatNT2; pYB4017 su9-mCherry |
| a | Alo1-GFP:His3MX6 fis1::HphNT1 |
| a | Alo1-GFP:His3MX6 fis1::HphNT1; pYB4017 su9-mCherry |
| a | Alo1-GFP:His3MX6 fis1::HphNT1 mks1::NatNT2; pYB4017 su9-mCherry |
| a | Alo1-GFP:His3MX6 fis1::HphNT1 rtg2::NatNT2; pYB4017 su9-mCherry |
| a | Oxa1-GFP:His3MX6 fis1::HphNT1 |
| a | Yme1-GFP:His3MX6 fis1::HphNT1 |
| a | Atm1-GFP:His3MX6 fis1::HphNT1 |
| a | Om45-GFP:His3MX6 fis1::HphNT1 |
| a | Por1-GFP:His3MX6 fis1::HphNT1 |
| a | fis1::HphNT1; pYB4019 su9-2×Kaede |

All yeast strains are BY4741 background with the genotype: his3Δ1 leu2Δ0 met15Δ0 ura3Δ0
